# Digistrophan, a New, Physiologically Standardised Cardiac Tonic

**Published:** 1910-12

**Authors:** 


**Digistrophan, a New, Physiologically Standardised Cardiac Tonic**

The frequently occurring statement in the literature that in the presence of strophanthus the objectionable cumulative effects of digitalis are, to a great extent, eliminated and the now experimentally confirmed fact that the joint action of these two similar, though not absolutely identically acting cardiac tonics, produces a superior therapeutic effect than can be obtained by each one separately, have led to the production of a digitalis-strophanthus preparation which is marketed under the name of digistrophan.

This product, which is manufactured in tablet form by a patented process excluding the otherwise unavoidable partial loss of active principles by volatilisation, is standardised exactly so that each tablet corresponds accurately to 1½ grains of digitalis leaves and ¾ grain of strophanthus seed of maximum physiological activity. Its originator, Dr. O. Boellke, Chief Physician of the Division for Internal Medicine, Municipal Hospital, Ratibor, Germany, illustrates the advantages of the new product in "*Therapie der Gegenwart,* 1910, No. 4, at the hand of eight clinical cases selected from a series of eighty-five which he has treated in the course of ten months. These comprise forty-four cases of organic defects of the cardiac valves, eighteen affections of the cardiac musculature, one of pericardial adhesion with cardiac displacement, three grave disorders of the cardiac action, due to thoracic stenosis, and nineteen of cardiac exhaustion in acute infectious diseases, particularly in typhoid.

The author gives data and curves showing accurately the prompt action of digistrophan in each of the eight cases and concludes from his experiments that the new cardiac tonic contains all the active principles of best digitalis leaves and strophanthus seed, while eliminating their objectionable cumulative effects, and that it can be relied upon to afford not only the highest stability and very convenient dosage but also unusual therapeutic activity.

